# Tyrosine kinase receptor RON activates MAPK/RSK/CREB signal pathway to enhance CXCR4 expression and promote cell migration and invasion in bladder cancer

**DOI:** 10.18632/aging.204279

**Published:** 2022-09-13

**Authors:** Junfeng Chen, Kejie Wang, Shazhou Ye, Xiangyu Meng, Xiaolong Jia, Youju Huang, Qi Ma

**Affiliations:** 1 Translational Research Laboratory for Urology, The Key Laboratory of Ningbo City, Ningbo First Hospital, The Affiliated Hospital of Ningbo University, Ningbo 315010, Zhejiang, China; 2 Ningbo Clinical Research Center for Urological Disease, Ningbo First Hospital, The Affiliated Hospital of Ningbo University, Ningbo 315010, Zhejiang, China; 3 College of Material, Chemistry and Chemical Engineering, Hangzhou Normal University, Hangzhou 310036, Zhejiang, China; 4 Comprehensive Urogenital Cancer Center, Ningbo First Hospital, The Affiliated Hospital of Ningbo University, Ningbo 315010, Zhejiang, China

**Keywords:** RON, CXCR4, CREB, cell migration, bladder cancer

## Abstract

Bladder cancer (BC) is one of the most lethal malignancies worldwide. The poor survival may be due to a high proportion of tumor metastasis. RON and CXCR4 are the key regulators of cell motility in BC, while the relationship between RON and CXCR4 remains elusive. In the present study, immunohistochemistry analysis of BC and adjacent normal tissues found that higher RON expression was positively correlated with CXCR4 expression. Inhibiting and replenishing RON level were used to regulate CXCR4 expression, observing the effects on migration and invasion of BC cells. Overexpression of RON reversed the inhibited cell migration and invasion following siCXCR4 treatment. Conversely, overexpression of CXCR4 restored the inhibition of cell migration and invasion caused by shRON. The activation of RON-MAPK/RSK/CREB pathway was demonstrated in BC cells under MSP treatment. Dual luciferase and CHIP assay showed that p-CREB targeted CXCR4 by binding to its CRE sequence. RON knockdown suppressed BC tumor growth in xenograft mouse tumors, accompanied by reduced expression of CXCR4. In conclusion, our data adds evidence that RON, a membrane tyrosine kinase receptor, promotes BC migration and invasion not only by itself, but also by activating MAPK/RSK/CREB signaling pathway to enhance CXCR4 expression.

## INTRODUCTION

Bladder cancer (BC) is the fourth most common malignant tumors in the United States with an estimated 81190 new cases and 17240 deaths by the end of 2018 [1]. Approximately 70%-80% of bladder cancers are non-muscle invasive (NMIBC), of which 50%-70% will recur and 4%-30% will progress to muscle-invasive disease [2]. Radical cystectomy is the standard treatment in the management of muscle-invasive bladder cancer (MIBC) [3], however, 15-90% disease will develop local recurrences or distant metastases even after radical cystectomy. 5-year survival rates are average only 27-66% for MIBC [3]. Thus, prevention of tumor invasion and metastases is a major challenge in BC. Although, various proteins such as transcription factors [4], adhesion molecules [5], extracellular matrix components [6], growth factors [7], protein tyrosine phosphatases and molecular chaperones [8] have been investigated in invasion and metastases of BC, the potential molecular mechanisms underlying bladder cancer invasion and metastases are still obscure.

Theoretically, the molecular pathogenesis of BC requires the deregulation of numerous signal transduction pathways. Thus, only key signaling events blocked by molecular targeted therapy may be useful to prevent bladder cancer invasion and metastases [9]. Identifying key signaling events is still challenging in BC and it is urgent to discover potential key molecular targets and related signal transduction pathways to provide effective therapeutic options in controlling BC invasion and metastases. Tyrosine kinase receptor RON, also known as macrophage stimulating protein receptor (MST1R), belongs to the MET proto-oncogene family. Studies have indicated that RON is highly expressed in various primary tumor samples including colon, breast, pancreas and bladder cancers, etc. [10–13]. At least four pieces of evidence suggested that altered expression of RON contributes to carcinogenesis and pathogenesis of BC. First, immunohistochemical staining revealed that RON was overexpressed in approximately 36% of primary BC tissues and several BC cell lines [13]. Second, RON expression was positive associated with the large tumor size, tumor stages, histological grade [14]. Third, co-expression of RON and epidermal growth factor receptor (EGFR) or MET were significantly associated with increased the risk of local recurrence, tumor invasion, and decreased patient survival [13]. Fourth, macrophage stimulating protein (MSP), the only known ligand of RON, which induces RON constitutive activation and signal transduction, was found in urine samples [13]. Thus, Aberrant expression of RON plays a key role in the carcinogenesis and malignancy of BC cells. Inhibition of RON pathway may have pharmaceutical potential for preventing BC cells invasion and metastases.

Chemokine gradients play an essential role in the directed cell movement in many normal and pathological processes. Chemokine receptor CXCR4 and its ligand CXCL12 are widely expressed in normal tissues and have been identified to involve in several biological and pathological processes such as trafficking and homeostasis of immune cells, attraction of cytokines presented at inflammatory sites, and promoting tumor cell migration and invasion [15]. Recent studies have shown that CXCR4 was related to cell survival, proliferation and metastasis in colon cancer, prostate cancer, lung cancer and pancreatic cancer, etc. [16–19]. CXCR4 overexpression was also detected in BC [20]. Multivariate analysis indicated that CXCR4 expression was positively associated with tumor size, clinical stage, and histological grade of BC [21]. These obtained data suggested that chemokine receptor CXCR4 is related to BC cells migration and invasion.

Though both RON and CXCR4 overexpression has been detected in BC and their expression levels are correlated with tumor size, histological grade, and clinical stage, the relationship between RON and CXCR4 is largely unclear. The purpose of this study is to reveal intrinsic the relationship between RON and CXCR4 and their roles in regulating bladder cancer migration and invasion. We believe that the results presented the study will help to increase understanding of the mechanism of RON mediated BC cell migration and invasion. Therefore, these results may be helpful for developing new tyrosine kinase receptor targeted therapy in BC.

## MATERIALS AND METHODS

### Main reagents

Mouse mAb zt/f2 specific to RON immunoglobulin, plexins and transcriptional factor (IPT) domain, MSP and rabbit antibody R5029 (specific to the RON C-terminal peptide) were kindly supplied by Prof Yao (State Key Laboratory for Diagnosis and Treatment of Infectious Diseases, Hangzhou, China). The primary antibodies against actin (cat. #4970S), Erk1/2 (cat. #4695S), phospho-Erk1/2 (cat. #4377S), Akt (cat. #9272S), phospho-Akt (cat. #9271S), p90 ribosomal S6 kinase 2 (RSK2) (cat. #5528S), phospho-RSK2 (cat. #11989S), CREB (cat. #9197S), phospho-CREB (cat. #9198S), E-cadherin (cat. #3195S) and vimentin (cat. #5741S) were all purchased from Cell Signaling Technology, Inc. (Danvers, MA, USA). Phospho-RON was bought from Santa Cruz Biotechnology (Santa Cruz, CA, USA). Anti-CXCR4 antibody (ab197203) was purchased from Abcam (Cambridge, MA, USA).

### Clinical tissue samples

The study included 106 BC patients’ specimens obtained during tumor surgical resection at the Ningbo First Hospital in the period of 2011-2014. Written informed consent was provided by all patients before sample collection. The patients had not received chemotherapy or radiotherapy before surgery. This study was also reviewed and approved by the Medical Ethics and Human Clinical Trial Committee of the Ningbo University school of medicine.

### Cell lines and cell culture

The human BC cell lines (5637, T24, TCCSUP, EJ, UMUC-3, BIU87 and J82) were purchased from National Collection of Authenticated Cell Cultures (Shanghai, China). The above cell lines were maintained in the Roswell Park Memorial Institute (RPMI) 1640 (Gibco; Thermo Fisher Scientific, Inc.) or minimal essential media (Gibco; Thermo Fisher Scientific, Inc.), supplemented with 10% fetal bovine serum (FBS) (Gibco; Thermo Fisher Scientific, Inc.) at 37° C with 5% CO2.

### Plasmid construction and lentiviral transfection

A short hairpin RNA oligonucleotide sequence (shRNA) for RON was used to knock down its expression. The sequence of RON shRNA was as follows: CCGGGAGGTCAAGGATGTGCTGATTCTCGAGAATCAGCACATCCTTGACCTCTTTTT. A scramble shRNA as a negative control was used as well. Lentiviral plasmids containing RON shRNA or scramble sequences were produced using 293T cells. siRNA for CXCR4: siCXCR4 (sense: 5′-GCAAGGCAGUCCAUGUCAUTT-3′, antisense: 5′-AUGACAUGGACUGCCUUGCTT-3′) and siRNA negative control: si-NC (sense: 5′-UUCUCCGAACGUGUCACGUTT-3′, antisense: 5′-ACGUGACACGUUCGGAGAATT-3′) were designed and purchased from Genepharma Biotechnologies (Shanghai, China). RON and CXCR4 overexpression constructs were designed and purchased from Genechem Co., Ltd. (Shanghai, China).

### Wound and healing assay

The cells were seeded in 6-well plates and were 90% confluent at the time of transfection. A 200 μl sterile pipette tip in a lengthwise stripe was used to scratch a clear line. After 24h, migration area was measured under a microscope and photographed. Experiments were repeated at least three times.

### Dual luciferase assay

CXCR4 promoter and its mutant luciferase reporter vectors, alongside with pRL-TK plasmid were transiently transfected into 5637 cells, separately. 24h later, serum-starved cells were stimulated for 30 min at 37° C by 5 nM MSP. Luciferase activity was assayed by using the Dual-Luciferase Assay System kit (Promega Bio Sciences, San Luis Obispo CA, USA) according to the manufacturer’s protocol.

### Chromatin immunoprecipitation (ChIP) assay

CHIP experiment was performed, with an EZ-ChIP assay kit (Millipore Technologies) following the manufacturer’s instructions and as described previously [22, 23]. In brief, 5637 cells were treated with MSP or mlgG for 30mins. All cells were cross-linked with 1% formaldehyde. Then genomic DNA and the proteins complex were isolated to generate chromatin DNA fragments size of 100 to 500bp. The Chromatin was subjected to immunoprecipitation (IP) using antibodies specific to p-CREB. After IP, DNA was extracted from the protein-DNA cross-links and then performed with PCR analysis. PCR was performed with the following pair of primers: 5′-CCGCGGCCAGAAACTTCA-3′(forward) and 5′-CCATGGTAACCGCTGGTTCT-3′(reverse).

### Immunohistochemistry and scoring

The methods were performed as described previously [14]. Paraffin-embedded specimens were cut into 4-μm sections. Then, the slices were deparaffinized, hydrated and treated with 0.3% H_2_O_2_ for 30 mins. The slides were cultured with rabbit anti-RON antibody Zt/f2 (1:200 dilution), anti-CXCR4 antibody (rabbit, 1:200 dilution) at 4° C overnight. The second anti-rabbit antibody (Abcam) was incubated at a dilution of 1:1000 for 1h at room temperature. Immunostaining was treated with EnVision System (DAKO Corp., Carpinteria, CA, USA) and stained with diaminobenzidine (DAB) supplied by the kit above.

Six fields were observed per slide and per view observed at 400× magnification. Immunostaining of RON and CXCR4 was scored on the staining proportion (on the scale of 0-4: 0: 0%-4%, 1: 5%-24%, 2: 25-49%, 3: 50-74%; 4:75-100%) and staining intensity (the intensity of RON membrane or CXCR4 Cytoplasm staining was defined as, 0, none; 1, weak; 2, intermediate; 3, strong). The two results cumulatively added to give a final score of 0-7. The final total score < 4 was considered as low expression, and ≥ 4 points was defined as a high expression.

### Transwell assay

A total of 1 ×10^5^ transfected 5637 or BIU87 cells in 200 μl serum-free RPMI 1640 medium per chamber respectively, were seeded into the upper chambers (8 μm pore-size, Corning Inc., NY, USA), and 600 μl RPMI 1640 medium containing 10% FBS were added to the lower chambers, respectively. After incubation for 24 h, the non-invaded cells on the top chamber were removed using cotton swabs, and the lower surface of the membranes were fixed with 800μl methanol for 30mins, stained in 0.1% crystal violet (Sigma-Aldrich, St. Louis, MO, USA) for 15mins, and washed with 1 × PBS twice. Finally, 5 randomly selected views of the invading cells on the bottom were manually counted with the CKX31 microscope (Olympus, Japan), with triplicates.

### Western blot analysis

The experiments were performed as previously described [22]. Proteins (60 μg/lane) were separated in 8% or 12% SDS-PAGE and transferred proteins to PVDF membranes. We then incubated the membrane with specific primary antibodies at 4° C overnight. After repeated washing with TBST, samples were incubated with the HRP-conjugated secondary antibodies for 1 hour at room temperature and detected by chemiluminescence (Pierce, Rockford, IL, USA).

### Xenograft tumor formation assay

All animal experimental procedures were approved (approval no.11198) by the Institutional Animal Care and Use Committee of Ningbo University. 4-to 6-weeks-old male BALB/c nude mice (weight, 15-18 g, 6 for each group) were purchased from the Shanghai SLAC Laboratory Animal Co., Ltd. (Shanghai, China). 1×10^7^ 5637 cells transfected with LV-shRON or LV-shNC were resuspended in 100μl Matrigel (BD Biosciences) and PBS (1:1) and subcutaneously inoculated into the right dorsal flanks of mice. After 18 d, tumor volume(mm^3^) was calculated as: Volume= 0.5 * length * width^2^ every 3 d during a 18d period. On day 36, the mice were sacrificed by intraperitoneal injection of 200 mg/kg pentobarbital sodium, death was verified by the absence of breathing and corneal reflex. Their xenograft tumors were photographed and subjected to western blot.

### Statistical analysis

Data of the study were analyzed by SPSS software (version 20; SPSS Inc., Chicago, IL, USA) and presented as the mean ± standard error (SEM). The Chi-squared test was used to analyze the relationship between RON and expression levels and the pathological clinic features. Transwell data and other data were analyzed using ANOVA or Student’s t test, respectively. The correlation between the RON and CXCR4 expression was assessed by using Pearson's correlation coefficient test. The statistical significance was determined by P <0.05.

## RESULTS

### RON is positively associated with CXCR4 expression in BC

**Figure 1:**
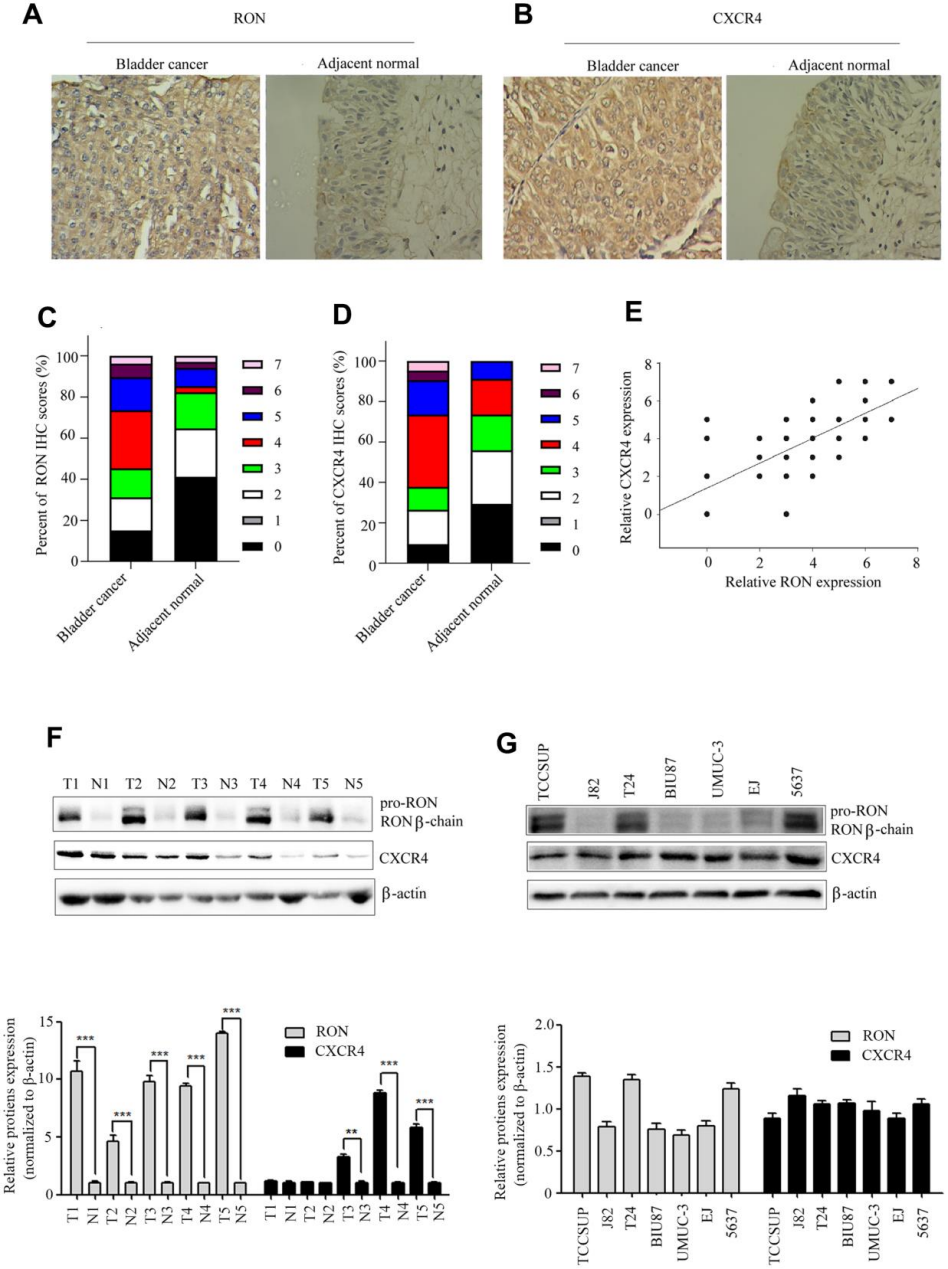
**High expression of CXCR4 is correlated with RON in BC.** Representative immunohistochemical staining for RON (**A**), and CXCR4 (**B**) protein expression in BC tumor and adjacent normal tissues. Average scores for RON (**C**), and CXCR4 (**D**) in BC tumor versus in adjacent normal tissues. The association with RON and CXCR4 expression in BC clinical samples was analyzed (**E**). RON and CXCR4 expression were detected by western blot in five representative BC tumor and corresponding adjacent non-tumor tissues (**F**), as well as in seven urothelial BC cells (**G**).

We first examined the expression status of RON and CXCR4 in tumor sections. The results showed that RON expression was significantly upregulated in 54.7% (58/106) of BC cases compared with the adjacent non-tumor tissues (6/34) and positively correlated with the number of tumor size, histological grade, and clinical stage and distant metastasis, as described in our published study (Figure 1A, 1B and Table 1 of ref. 24). In this study, a similar trend in CXCR4 expression (65/106 with highly expressed) was also revealed as shown in (Figure 1B, 1D), and Table 1. The RON expression was clearly correlated with expression of CXCR4 in BC tissues (*P*<0.01; Pearson's coefficient, r=0.726) (Figure 1E). To further evaluate the correlation between CXCR4 expression, five representative cancer specimens with relatively high RON expression level were assayed by western blotting. The cancer tissues with higher levels of RON expression also tended to have higher CXCR4 expression (Figure 1F), indicating that high CXCR4 expression might be related to RON expression in the pathogenesis of bladder cancer. Moreover, RON expression was highly detected in TCCSUP, 5637 and T24 cell lines, while barely found in other four cancer cell lines (Figure 1G). All of the BC cell lines expressed CXCR4 protein (Figure 1G). These results indicate that co-expression of RON and CXCR4 is a common event in BC, and RON expression is positively associated with CXCR4.

**Table 1:** Relationship between the expression of CXCR4 and clinical pathological features of bladder cancer.

**Variables**	**N**	**CXCR4 expression**		**χ^2^**	***P***
		**Low**	**High**		
Tissue				12.547	0.000
All tumor	106	41	65		
Paracancerous	34	25	9		
Gender				2.468	0.116
Male	87	40	47		
Female	19	5	14		
Age				1.7335	0.118
<70	51	25	26		
≥70	55	20	35		
Number of tumor nodules				4.422	0.0035
Single	63	32	31		
Multiple	43	13	30		
Distant metastasis				6.920	0.009
M0	90	43	47		
M1	16	3	13		
Pathological stage				3.743	0.041
T_is_ - T_1_	42	24	18		
T_2_ - T_4_	64	21	43		
Histological grading				6.774	0.021
Urothelial carcinoma, grade I	10	10	0		
Urothelial carcinoma, grade II	41	24	17		
Urothelial carcinoma, grade III	55	11	44		

T, tumor; M, metastasis.

### CXCR4 is involved in the RON-regulated cell migration and invasion *in vitro*

**Figure 2:**
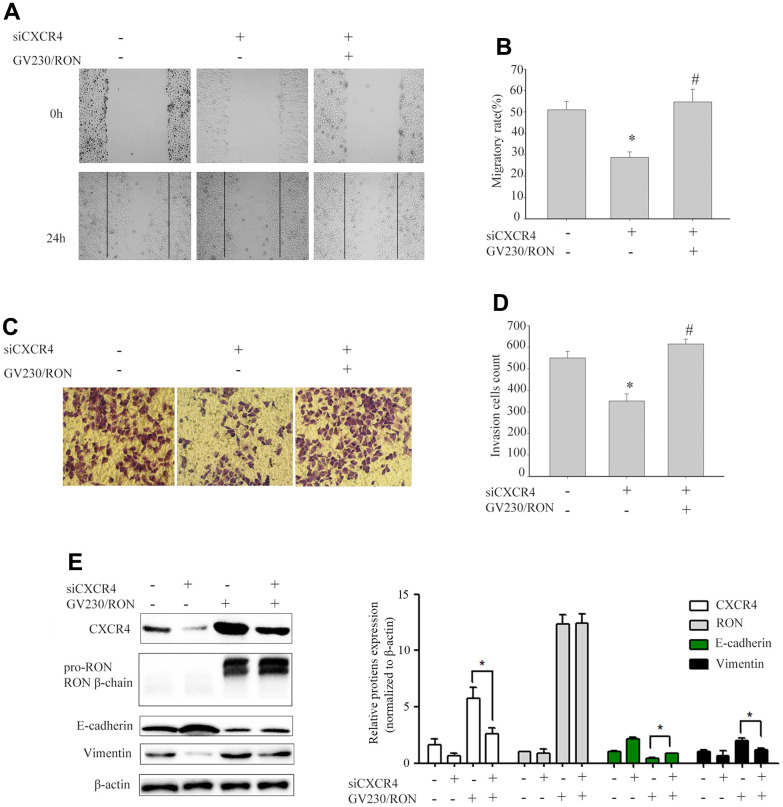
**Ectopic expression of RON reverses the reduction of CXCR4 and the inhibition of cell migration and invasion by siCXCR4.** After BIU87cells were transfected with siCXCR4 in the absence(-) or presence(+) of GV230/RON, wound healing assay for analysis of the cell migration (**A**, **B**), transwell invasion assay analysis of the cell invasion (**C**, **D**) and Western blot analysis of CXCR4, RON, E-cadherin and Vimentin expression (**E**). After down-regulation of CXCR4 with siCXCR4, the CXCR4 expression was significantly reduced, cell migration and invasion were also inhibited. However, overexpression of CXCR4 in BIU87 cells co-transfected with GV230/RON reversed CXCR4-reduced protein levels, cell migration and invasion. Data are shown as mean ± SEM from one of three experiments with similar results. *P < 0.05, versus absence of GV230/RON and siCXCR4 treatment, *^#^P* < 0.05, versus siCXCR4 treatment.

To explore whether CXCR4 was involved in RON- regulated cell migration and invasion, we first explored the role of CXCR4 in cell migration and invasion. After successfully decreasing CXCR4 expression levels by siCXCR4 treatment, cell migration and invasion was inhibited (data not shown). Then, using GV230/RON vector containing the RON coding sequence increased the expression of RON in BIU87 cells, to determine whether overexpression of RON could counteract the reduction of CXCR4 and the inhibition of cell migration and invasion by siCXCR4. We found that ectopic expression of RON reversed the suppression of CXCR4 and the reduction of cell migration (Figure 2A, 2B) and invasion (Figure 2C, 2D). In addition, siCXCR4 treatment significantly induced downregulation of the epithelial marker E-cadherin and upregulation the mesenchymal markers vimentin, whereas, these were reversed by the combined treatment with siCXCR4 and GV230/RON (Figure 2E).

### Overexpression of CXCR4 restores the inhibition of cell migration and invasion caused by RON reduction

**Figure 3:**
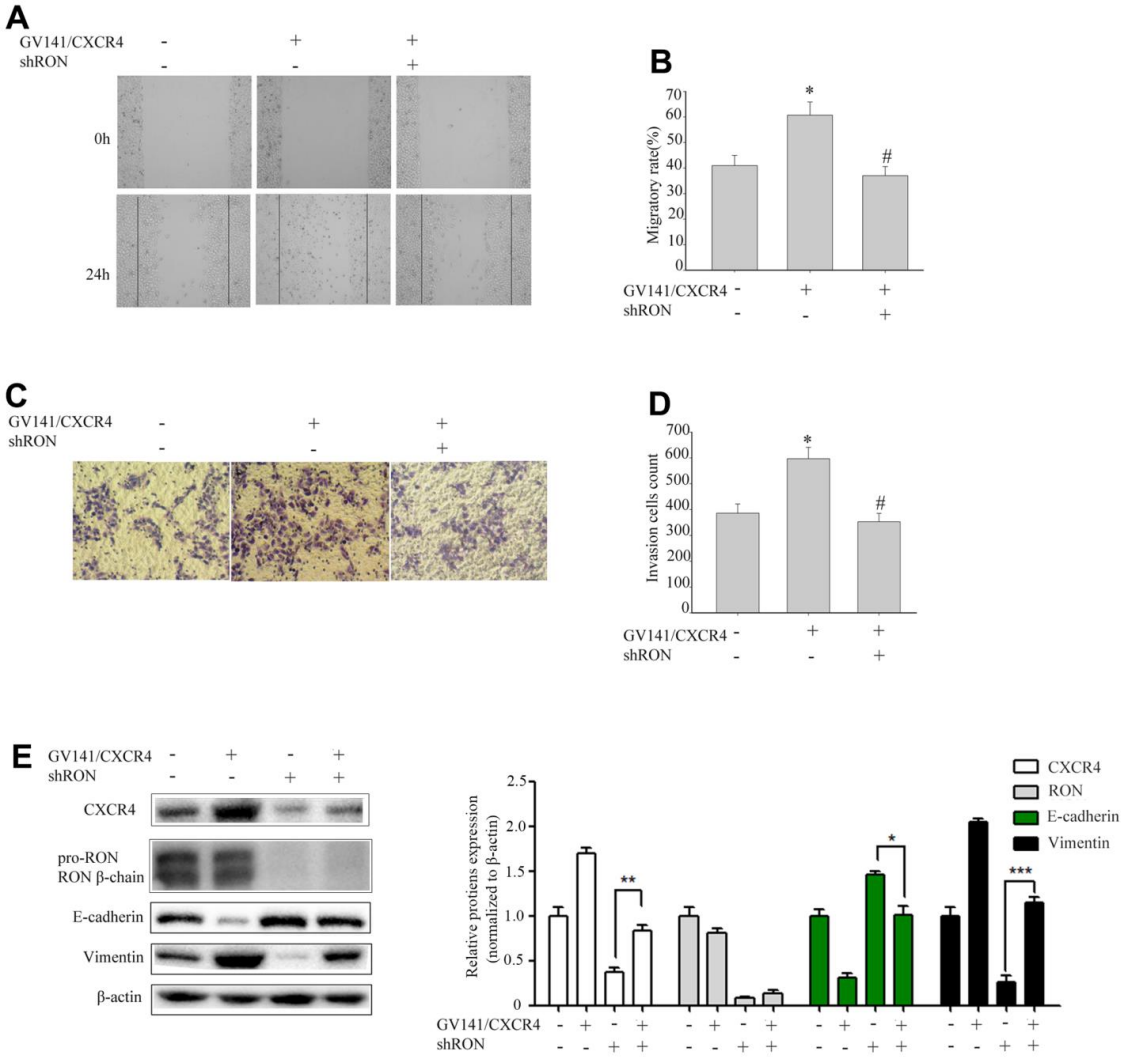
**Overexpression of CXCR4 restores the inhibition of cell migration and invasion caused by RON reduction.** 5637 cells GV141/CXC4 or control were added to shRON (+) or shNC (-). The wound healing assay was performed to evaluate the capability of cell migration (**A**, **B**). The transwell assay was used to detect the cell invasion ability (**C**, **D**). Cell extracts were subjected to Western blot analysis using the indicated antibodies to CXCR4, RON, E-cadherin, and vimentin, respectively (**E**). Data are shown as mean ± SEM, from one of 3 independent experiments. *P<0.05, versus absence of GV141/CXCR4 and shRON treatment, *^#^P* < 0.05, versus GV141/CXCR4 treatment.

We further evaluated the GV230/CXCR4 vector in 5637 cells to determine whether overexpression of CXCR4 could restore the inhibition of cell migration and invasion caused by RON reduction. shRON treatment could significantly reduce CXCR4 expression, cell migration and invasion were also significantly inhibited (data not shown). 5637 cells were transfected with an expression vector containing CXCR4 cDNA overexpressing CXCR4. The ectopic expression of CXCR4 in the 5637 cells promoted cell migration (Figure 3A, 3B) and invasion (Figure 3C, 3D), led to a significant increase of vimentin expression, and a corresponding reduction of E-cadherin expression (Figure 3E). Whereas these were restored by the combined treatment with shRON and GV230/CXCR4. These findings suggest that RON probably promotes BC cell migration and invasion by increasing CXCR4 expression that modulates the key EMT regulating factors.

### MSP induces RON phosphorylation and its specificity in activation of MAPK/RSK/CREB signaling

**Figure 4:**
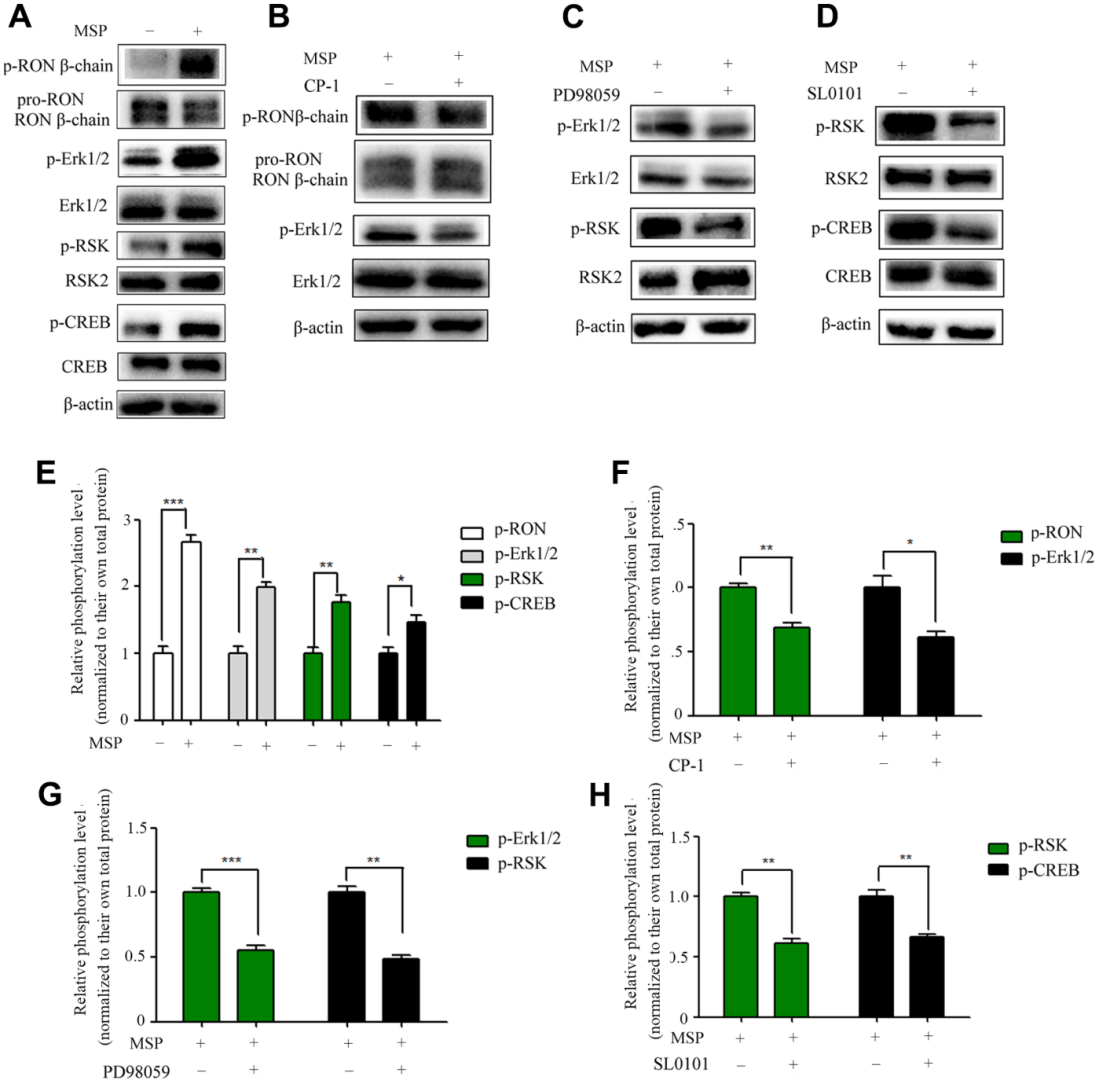
**Effect of MSP on RON phosphorylation and activation of downstream signal molecules.** 5637 cells (2 × 10^6^ cells per 60-mm culture dish) were incubated in RPMI-1640 containing 1% FBS overnight and then stimulated for 30 mins with MSP (5nM). Western blot analysis measured the effects of MSP on the phosphorylation levels of RON, Erk1/2, RSK2 and CREB (**A**). 5637 cells (2 × 10^6^ cells per dish) in RPMI-1640 with 1% of FBS were first treated with Small chemical inhibitors specific to RON (CP-1, 300 nM) (**B**), Erk1/2 (PD98059, 150 μM) (**C**), and RSK (SL0101, 50μM) (**D**) for 12 h, respectively, followed by stimulation with MSP (5nM). Cells were collected 30 mins after stimulation. Phosphorylated RON, Erk1/2, RSK2 and CREB were directly detected by Western blot analysis, respectively. Non-phosphorylated proteins were also determined as the loading controls. (**E**–**H**), Quantitative analysis, *P<0.05, **P<0.01 and ***P<0.001 VS. normalized to their own total protein group. Data are shown as mean ± SEM, from one of 3 independent experiments.

Overexpression of RON promotes cell migration and invasion in BC [13, 14]. However, the main RON downstream signal molecules to these changes are unclear. To determine these molecules, we performed MSP to induce RON activation. As shown in Figure 4A, MSP was capable of inducing phosphorylation of RON, Erk1/2 (p44/42), RSK2 and CREB. These results suggested that MSP stimulation induces RON activation resulting in phosphorylation of downstream signal proteins. As expected, the effect of MSP was reversed when RON, ERK1/2, and RSK2 signals were suppressed by CP-1 (Figure 4B), PD98059 (Figure 4C), and SL0101 (Figure 4D), respectively. These results indicated the importance of the RON-Erk/RSK/CREB pathway in regulating cell migration and invasion in bladder cancer.

### RON activation upregulates CXCR4 promoter activity and promotes CREB binding to CXCR4 gene promoter

**Figure 5:**
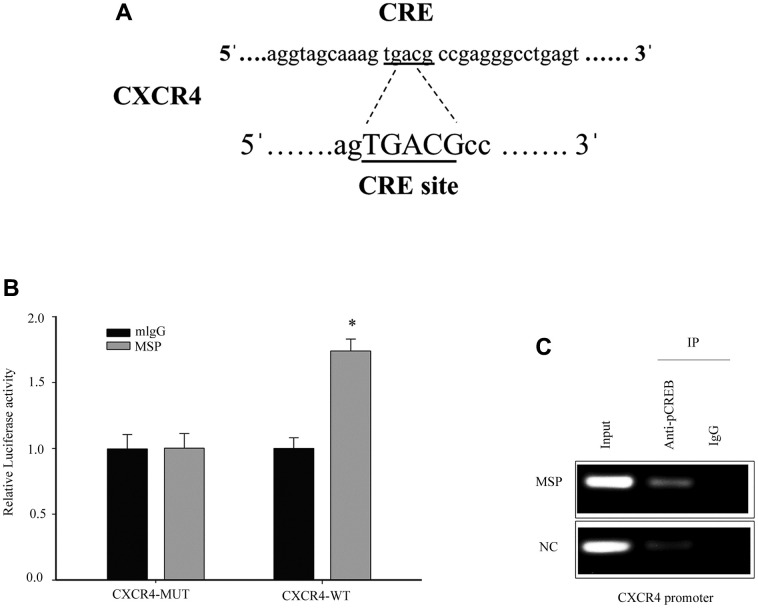
**Induced p-CREB promotes CXCR4 transcription by targeting the CXCR4 promoter.** The potential CREB targeting site in CXCR4 promoter (**A**). Cells were first transfected with CXCR4 promoter driven luciferase reporter for 24h and then incubated with 5 nM MSP for 30 mins. Dual-Luciferase Reporter Assay System was used to detect the luciferase activity (**B**). After activation of MSP, Chromatin immunoprecipitation (ChIP)-PCR was performed to show that p-CREB bound to the putative promoter of CXCR4 in 5637 cells (**C**). Data were presented as mean ± SD from three independent experiments. *P < 0.05 versus mlgG treatment. WT: wild type; Mut: mutant type.

The transcription factor cAMP responsive element binding protein (CREB) is able to bind to CXCR4 cAMP-responsive element (CRE) site. Previous studies have demonstrated that Phospho-CREB (p-CREB) can upregulate CXCR4 promoter transcriptional activation [24]. Thus, we further studied CXCR4 as a potential target (Figure 5A). To detect the effect of CREB on the cellular CXCR4 expression, we constructed and transfected the luciferase reporter driven by the CXCR4 promoter into 5637 cells. MSP treatment resulted in a significant increase (1.7 folds) in the activity of the CXCR4 promoter-driven luciferase reporter (Figure 5B). To investigate if the transcription factor CREB was responsible for RON activation induced upregulation of CXCR4 transcription, a ChIP assay was performed and we found that p-CREB directly bonded to the CXCR4 promoter region containing CRE binding sites (Figure 5C). Thus, our results demonstrate that RON activation promoted p-CREB binding to CXCR4 gene promoter and regulated CXCR4 transcription in response to changes in cell migration and invasion.

### Knockdown of RON inhibits bladder cancer cell growth and reduces CXCR4 expression *in vivo*

**Figure 6:**
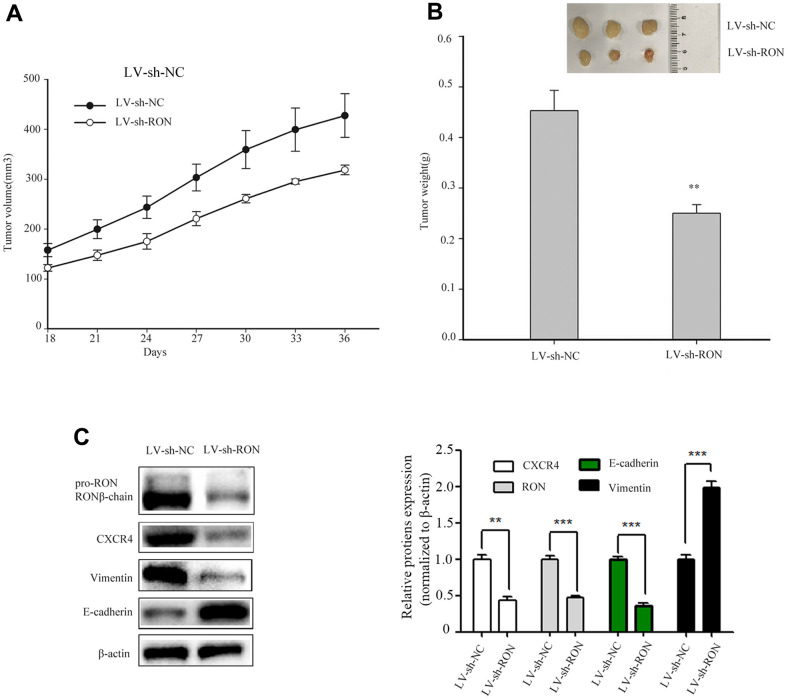
**Down-regulation of RON inhibits tumorigenesis *in vivo* and reduces CXCR4 expression *in vivo*.** Representative images of xenograft tumor formation were shown in the LV–shRON group and LV–shNC group in nude mice. RON knockdown reduced the tumor volume and tumour weight in nude mice. The tumor volume growth curve was significantly inhibited in the LV–shRON group (**A**), and the average tumor weights were markedly lower, as compared with those in the LV–shNC group (**B**), Furthermore, the CXCR4, E-cadherin and Vimentin proteins expression was significantly decreased or increased compared with controls (**C**). All data are shown as means ± SEM, **P < 0.01 versus scramble.

To examine the tumorigenic ability of RON *in vivo*, we constructed shRNA sequences targeting the RON vector into lentivirus (LV). 5637 cells expressing LV–shRON or LV–shNC were injected into the right flanks of nude mice, and tumor volumes were measured every 3 days after cell injection for 18 days. From the tumor growth curve, tumors in LV–shRON group grew slower than those in LV–shNC group (Figure 6A). All the mice were euthanized at 36 days after injection, and the xenografts were excised. The average tumor weight in the LV–shRON group was significantly decreased compared with the LV–shNC group (*p*<0.05, Figure 6B). The expressions of RON, E-cadherin, vimentin and CXCR4 in mice tumors were determined by Western blot. The RON, CXCR4 and vimentin protein expressions in RON shRNA group were markedly lower while E-cadherin was higher than those in the LV–shRON group (Figure 6C). These data suggested that RON knockdown can inhibit CXCR4 expression *in vivo*.

## DISCUSSION

BC is still one of the most lethal cancers. When BC progresses to muscle invasion and metastases, patients' survival rate will be dramatically decreased. Despite many studies have been conducted, the underlying mechanisms of BC progression are still largely unclear.

**Figure 7:**
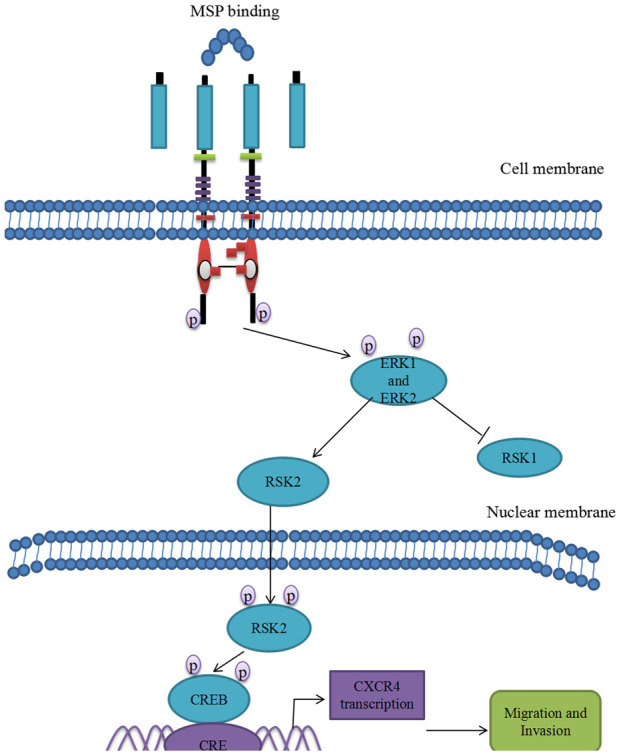
**Proposed mechanistic scheme of RON promoting tumor invasion in bladder cancer.** MSP stimulation induces RON dimerization by binding the sema domain, which stimulates the Erk1/2-RSK signal transduction pathway regulating RON-mediated activities such as cell growth and invasiveness. Activated ERK1 and ERK2 also stimulate RSK2 and promote RSK2 nuclear translocation, which induced CREB phosphorylation. Phospho-CREB binds cAMP-responsive element (CRE) site of CXCR4, thereby inducing its transcription and over-expression.

In the present study, we found that overexpression of RON and CXCR4 was positively associated with the aggressive behaviors in bladder cancer, which supports the hypothesis that RON related signaling pathways play a vital role in bladder cancer invasion and metastasis. Our results are consistent with previous findings on the significance of RON and CXCR4 in bladder cancer [13, 21]. The purpose of this study was to determine whether RON regulates the expression of CXCR4, a chemokine receptor that plays a critical role in tumor cell invasion and metastasis [25]. In the present study, the evidences from both BC tissues and BC cells had clearly indicated that RON activation upregulate CXCR4 expression. Firstly, immunohistochemical staining analysis and Western blotting experiments suggested that the expression of CXCR4 was higher in RON positive BC tissues compared with adjacent non-carcinoma tissues (Figure 1 and Table 1, ref. 24 and Figure 1 and Table 1). Moreover, the expression levels of RON and CXCR4 exhibited a marked correlation with the progression of bladder tumor stage. Secondly, transfection of RON into RON-negative BIU87 cells significantly increased CXCR4 expression, meanwhile, BIU87 cells migration and invasion was reduced with siCXCR4, and this effect was reversed by ectopic administration of RON (Figure 2). Correspondingly, silencing of RON dramatically decreased CXCR4 expression, cell migration, and invasion in 5637 cells, and the ectopic expression of CXCR4 could restore the inhibition (Figure 3). Thirdly, knockdown of RON could suppress tumor formation and result in the lower expression of CXCR4 in nude mice tumorigenesis (Figures 6, 7). These findings further confirmed that RON regulates CXCR4 expression.

RON signaling has been studied in the development and treatment of tumors for over 20 years. Aberrant RON activation featured by ligand binding [26], overexpression of RON protein [27], oncogenic variant production [28] and kinase domain point mutations [29] has been found in many types of cancers. In our previous study, RON overexpression was detected in 40% BC tissues [14]. We also used antibody specific to RON oncogenic variant RON160 for immunohistochemical staining in BC tissues, but failed to get positive staining (data not shown). It was reported that detectable urinary MSP was found in transitional-cell BC patients and RON regulated invasive growth and was an independent predictor of distant metastasis in BC [13]. Thus, RON continuous activation may exist in some BC patients due to RON overexpression or ligand induced activation.

RON activation is featured by auto phosphorylation at Tyr1238 and Tyr1239 in its kinase domain [30–32]. Phosphorylation of these regulatory residues further phosphate Tyr1353 and Tyr1360 in the C-terminal docking site [30–32]. The docking site involves in downstream signal transduction such as RAS-MAPK and PI3K-AKT pathways [30–33]. These signaling pathways contribute to increasing cell proliferation, epithelial to mesenchymal transition (EMT), migration and invasion [34]. In our previous research, we have found that RSK2 is the key molecule in RON mediated cancer cell migration and invasion [35]. Here we further demonstrated that CREB, a downstream signal protein of RSK2 [36], was activated by this signaling pathway. The phosphorated CREB binds to CXCR4 promoter and promotes CXCR4 transcription.

The primary tumor developed to a disseminated metastatic disease is a complex process. Emerging evidences suggested that chemokines and their receptors play an important role in the metastatic process. CXCR4 and its ligand, CXC chemokine ligand 12 (CXCL12), have been shown to be key regulators of tumor invasion and metastasis [37]. Blocking of CXCR4 function by a monoclonal antibody clearly inhibited cancer cell proliferation, motility and invasion [38]. Retz et al. [38] reported CXCR4 is expressed in bladder cancer cell lines and cancer tissues in a tumor stage-dependent manner, which is consistent with our data [Figure 1 and Table 1]. Shen et al. reported that CXCR4 is required for CXCL12-induced cell invasion and depletion of CXCR4 in bladder cancer impairs cell invasion [39]. As reported by Basti et al., CXCR4 expression was positively correlated with tumor stage and grade in BC tissues [40]. These findings suggested that CXCR4 plays an important role in the regulation of BC migration and invasion. The mechanism of CXCR4 overexpression induced cell metastasis has been studied in several types of cancer. In hepatic cancer, TCF12 promoted CXCR4 expression through activation of MAPK/ERK and PI3K/AKT signaling pathways [41]. In breast cancer, PI3-kinase, act as key regulatory components involved in CXCR4 induced chemo-invasion [42]. In the glioma, LRRC4, an onco-suppressive gene, inhibited CXCR4-induced cell invasiveness by reducing ERK1/2 and Akt signaling [43]. Here we reported overexpression or activation of tyrosine kinase receptor RON promotes CXCR4 expression through ERK/RSK2/CREB pathway (Figure 4). It has been shown that the promoter region of CXCR4 contains some potential transcription factor binding sites such as NF-kB binding sites [44], NRF-1 sites [45] and CREB sites [24]. In this study, dual luciferase assay was performed to verify that CREB binds to CXCR4 gene promoter (Figure 5B). With p-CREB as a target, CHIP assay showed a physical interaction between p-CREB and the CXCR4 promoter when 5637 cells were stimulated with MSP, which activated RON and upstream ERK/RSK2/CREB (Figure 5C), providing direct evidence that RON act as a key regulator in promoting CXCR4 expression in BC cells.

In conclusion, we firstly revealed the intrinsic relationship between tyrosine kinase receptor RON and chemokine receptor CXCR4. At least three pieces of evidence verified that RON overexpression or activation enhanced expression level of CXCR4. First, RON and CXCR4 overexpression was highly positively related in clinical BC samples. Second, overexpression RON or activation RON prompted CXCR4 overexpression and blocking RON or inhibiting RON deregulated CXCR4 expression. Third, the potential pathway involving in RON regulation of CXCR4 was MAPK/RSK/CREB. RON activation promotes p-CREB binding to CXCR4 promoter. Thus, RON activation not only promotes BC invasion and migration by itself, but also enhances chemokine receptor CXCR4 expression, which further increases the ability of BC invasion and migration (Figure 7). Blocking RON signal pathway may provide a therapeutic opportunity to prevent BC invasion and subsequent metastases.
